# Effects of the Wheat Crab Model and the Pond Culture Model on the Growth, Metabolism and Intestinal Microbiota of the Chinese Mitten Crab (*Eriocheir sinensis*)

**DOI:** 10.3390/microorganisms13102396

**Published:** 2025-10-19

**Authors:** Min Yang, Jun Ling, Tong Li, Chengchen Yu, He Jiang, Tingshuang Pan

**Affiliations:** Anhui Key Laboratory of Aquaculture and Stock Enhancement, Fishery Institute of Anhui Academy of Agricultural Sciences, Hefei 230041, China; yangmin831001@163.com (M.Y.); fisherlling@163.com (J.L.); little_li_tong@163.com (T.L.); ccyu549@163.com (C.Y.); hfjianghe@sina.cn (H.J.)

**Keywords:** Chinese mitten crab, growth, metabolism, intestinal microflora, wheat-crab model

## Abstract

This study investigated the influence of two distinct aquaculture systems, namely, the wheat-crab model (WCM) and pond culture (PC) model, on the growth, physiological well-being, and gut microbial structure of Chinese mitten crabs (*Eriocheir sinensis*). A total of 120 adult crabs were randomly selected from the two systems: 60 crabs from the WCM, including 30 females and 30 males, and 60 crabs from the PC model, also including 30 females and 30 males. The gonadosomatic index of female crabs in the WCM was notably higher than that of the other groups, while the hepatopancreatic index was significantly lower. Significant variations were not observed in final weight, fullness, or muscle yield between the WCM and PC groups. Biochemical evaluations indicated no substantial differences in antioxidant capabilities between the two systems; however, female crabs demonstrated increased critical antioxidant enzyme activity, such as for catalase and superoxide dismutase, and significantly elevated cholesterol levels. Additionally, the expression of the genes *IL*, *ProPO*, and *Keap1* was significantly higher in the WCM group than the PC group, whereas the expression of *ALF2*, *Myd88*, and *CncC* did not significantly differ between the two cultivation methods. Non-targeted metabolomics analysis revealed notable distinctions in metabolite profiles between the two systems. Moreover, the WCM facilitated an increase in beneficial Firmicutes bacteria while reducing potentially harmful microorganisms, suggesting improved immune function in crabs raised under the WCM. In summary, female crabs reared in the WCM matured earlier and exhibited slightly better health conditions compared to those grown in the PC model.

## 1. Introduction

The Chinese mitten crab (*Eriocheir sinensis*) is a significant freshwater crustacean species. Due to its high nutritional content and economic value, it is extensively farmed across many provinces in China [1]. With the development of large-scale crab culturing in recent years, this industry has been challenged by various diseases associated with the culture environment. Thus, it is crucial to create more sustainable *E. sinensis* breeding models.

Some pond culture (PC) models in Anhui Province have been transformed from farmland. Although the PC model can enhance economic benefits in the short term, with the strengthening of China’s food security policies [2], such aquaculture methods that occupy basic farmland are facing the risk of being rectified or even banned. Meanwhile, the drawbacks of the PC model have become increasingly prominent. The PC model relies on artificial feeding of compound feed, with a large accumulation of leftover feed and excrement, which can easily cause eutrophication of water bodies [3], leading to algal blooms and insufficient dissolved oxygen, thereby increasing the risk of diseases in Chinese mitten crabs and reducing the farming benefits. Against this backdrop, the WCM has emerged as an innovative approach, using the wheat intercropping period to create a good growing environment for crabs, and exhibits several advantages over the pond model, including reduced fertilizer use, reduced pesticide use, efficient land and water use, resource recycling, and improved economic efficiency. Previous studies have shown that the aquaculture environment of crabs affects their growth, nutrition [4], health status [5] and intestinal microbial community composition [6], among other factors. Many studies have shown that rice-crab culture can affect the nutrient composition of *E. sinensis*, improve the ecological environment and enhance economic benefits [7]. Although the WCM farming method was successfully implemented in China in 2022 and 2023, how this system affects the growth, metabolism and intestinal microbiota of *E. sinensis* remains a blank.

Similar to other crustaceans, Chinese mitten crabs lack a typical adaptive immune system [8], and their defense system primarily consists of an innate immune response that include various endogenous immune system expression or inducible factors [9] and the activity of various immune-related enzymes (e.g., SOD, GSH-Px, MDA, and CAT) [10,11]. Consequently, when these crabs are subjected to biological or abiotic stressors, the cellular equilibrium can be disturbed, leading to the production and buildup of reactive oxygen species (ROS) [12,13]. An imbalance between antioxidant defenses and ROS levels can lead to the increased production of oxygen ions, free radicals, and peroxides, which subsequently induces oxidative stress [14]. Therefore, fluctuations in various antioxidants in serum and hepatopancreas reflect the health of crabs [15]. Previous studies have found that *TLR*s may be essential in the antibacterial and anti-fungal immune response of crabs, improving the innate immune response [16]. In amphibians, overexpressed *MyD88* triggered the NF-κB promoter, indicating that *MyD88* may play an important role in inflammatory responses [17]. Therefore, it is possible to gain a deeper understanding of the adaptation mechanisms and immune defense strategies of *E. sinensis* within the WCM by analyzing the expression patterns of immune-related factors.

The gut microbiota plays a vital role in the digestion, nutrient absorption, and immune function of crustaceans [18,19,20]. Alterations in the gut microbial community can significantly affect host health and disease resistance. Metabolomics analysis, particularly non-targeted metabolomics, enables a holistic profiling of small-molecule metabolites, revealing metabolic changes induced by environmental factors or dietary interventions [21]. By integrating gut microbiota and metabolomics analysis, this study provides a deeper understanding of the impact of the WCM on the physiological and immune status of crabs, providing valuable insights for sustainable aquaculture practices.

To address this knowledge gap, we conducted a study in Dangtu County, Anhui Province, analyzing the growth performance, biochemical parameters, metabolome, and gut microbiota of crabs under the WCM model. This research aims to provide a robust scientific foundation for assessing the viability and promoting the adoption of the environmentally friendly WCM in *E. sinensis* aquaculture.

## 2. Materials and Methods

All animal procedures in this study were conducted in accordance with relevant guidelines and regulations, and were specifically approved by the Animal Ethics Committee of the Fishery Institute, Anhui Academy of Agricultural Sciences (Approval No. AAAS2023-9 on 15 March 2023).

### 2.1. Sites and System Description

**Figure 1:**
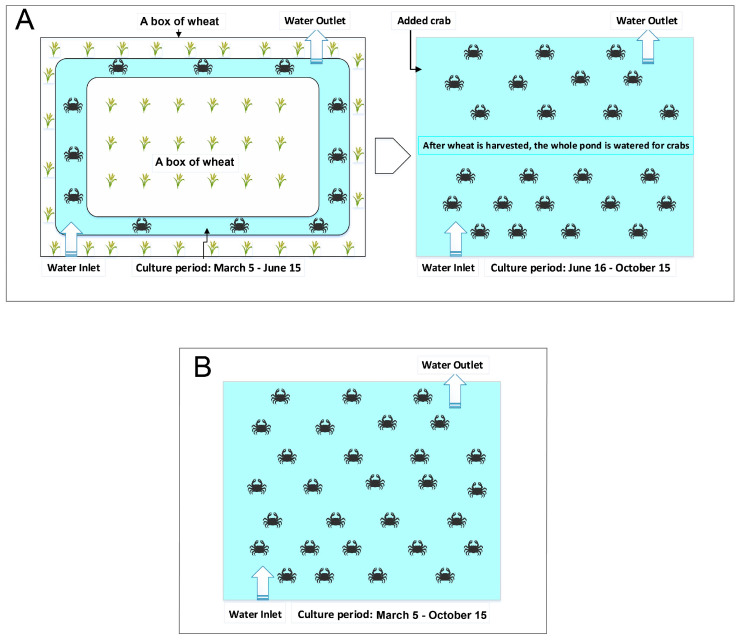
Schematic diagram of the breeding model. (**A**) the crab-wheat model. (**B**) the pond culture model.

The experiment was conducted in a wheat-crab breeding demonstration site (Guangfeng Aquaculture Family Farm) in Dangtu City, Anhui Province, China (longitude: 118.58°, latitude: 31.46°). Chinese crab larvae were obtained from a Guangfeng aquaculture family farm. The WCM had an area of 32 × 667 m^2^, and trenches (1.2 m × 0.8 m, width × depth) were dug around each plot to breed the Chinese mitten crabs (Figure 1). The wheat growing season was from November 2022 to May 2023, and field operations included sowing, fertilizing, weeding, and harvesting. After the wheat was harvested, the straw was removed, the land was tilled, and water was injected into the soil to plant grass. After 15 days, the density of *E. sinensis* was 1200/667 m^2^ (60 ± 5 g). The pond culture (PC) model had an area of 30 × 667 m^2^, grass was planted in December, and crabs were added to the pond in March at a density of 1500/667 m^2^ (10 ± 2 g). A 35 cm-high fence was constructed around the two experimental plots to prevent the crabs from escaping. Water inlets and outlets were also installed in each plot. Each field was equipped with two water wheel aerators, each with a power of 2.20 kW/h. During the experiment, 4% of the crab body weight was fed with commercial pellet feed containing 40% crude protein and 4% crude fat (provided by Chengdu Tongwei Co., Ltd. in Chengdu, China) twice a day, at 6:30 a.m. and 3:00 p.m., and minor adjustments were made according to the remaining feed volume. The water management, feeding schedule, and daily maintenance for the two breeding plots were identical.

### 2.2. Sample Collection

On 15 October, the crabs were ripe. Sixty crabs (30 females [C♀] and 30 males [C♂]) were randomly selected from the WCM and sixty crabs (30 females [C♀] and 30 males [C♂]) were randomly selected from the PC model for sampling. Five minutes after cryoanesthesia, hemolymph was collected from the bottom of the third leg using a sterile syringe. Hemolymph was injected into sterile and enzyme-free centrifuge tubes, refrigerated overnight at 4 °C, and then centrifuged at 12,000× *g* for 8 min at 4 °C. The supernatant was stored at −80 °C for subsequent biochemical analysis. The hepatopancreas, muscles and gonads were dissected and weighed to calculate the performance indicators. Hepatopanpancreas and muscle samples were stored at −80 °C for further metabolomics and genetic quantitative examination. In addition, intestinal chyme specimens were collected on a sterile operating table and stored at −80 °C for microbial community analysis.

After a 24 h fasting period, the fullness (FNS), hepatopancreas index (HSI), gonadosomatic index (GSI), and muscle yield (MYI) of the crabs were determined using the following equations:

FNS (g/cm) = (whole body weight) (g)/(carapace length)^3^

HSI (%) = hepatopancreas weight × 100/whole-body weight

GSI (%) = gonad weight × 100/whole-body weight

MYI (%) = muscle weight × 100/whole-body weight

### 2.3. Chemical Analysis

The hepatopancreas samples were weighed and homogenized in 10 volumes (*v*/*w*) of ice-cold 0.85% saline solution. The resulting homogenate was centrifuged at 12,000× *g* for 10 min at 4 °C, after which the supernatant was collected and stored at −80 °C for subsequent analysis of antioxidant parameters. The concentrations of malondialdehyde (MDA), total superoxide dismutase (SOD), glutathione peroxidase (GSH-Px), catalase (CAT), alanine aminotransferase (ALT), alkaline phosphatase (ALP), triglycerides (TG), total protein (TP), total cholesterol (CHO), and amylase (AMY) were measured in the hepatopancreas, muscle, and hemolymph. All measurements were conducted using diagnostic reagent kits (provided by Jiancheng Co., Ltd., Nanjing, China) following the manufacturer’s protocols. There were a total of 6 samples, and each sample was tested three times.

### 2.4. Gene Expression Analysis

Total RNA was extracted from approximately 50 mg of hepatopancreas tissue using TRIzol reagent (Invitrogen, Waltham, MA, USA), followed by purification with the Ultrapure RNA Kit (DNase I) (CW0597S, CWBIO, Taizhou, China) according to the manufacturer’s instructions. RNA concentration and purity were measured using a NanoDrop 2000 spectrophotometer (Thermo Scientific, Waltham, MA, USA), and integrity was verified by 1% agarose gel electrophoresis.

First-strand cDNA was synthesized from 1 µg of total RNA using the TransScript One-Step gDNA Removal and cDNA Synthesis SuperMix (AE311, TransGen Biotech, Beijing, China) in a 20 µL reaction system containing 10 µL of 2 × TS Reaction Mix, 1 µL of RT/RI Enzyme Mix, 1 µL of gDNA Remover, 1 µL of Random Primer (10 µM), and RNase-free water. The reaction was incubated at 42 °C for 15 min, followed by 85 °C for 5 s.

Quantitative real-time PCR (qPCR) was performed on a LightCycler 96 system (Roche, Basel, Switzerland) using TransStart Green qPCR SuperMix (AQ131-01, TransGen Biotech, Beijing, China). Each 20 µL reaction contained 10 µL of 2 × SuperMix, 0.5 µL each of forward and reverse primers (10 µM), 2 µL of diluted cDNA, and 7 µL of RNase-free water. The thermal cycling conditions were as follows: initial denaturation at 94 °C for 30 s; 45 cycles of 94 °C for 5 s and 61 °C for 35 s; followed by a melting curve analysis (95 °C for 10 s, 65 °C for 60 s, and 97 °C for 1 s). All samples were run in triplicate, including no-template controls.

Primers for target genes (*IL*, *30*, *ProPO*, *Keap1*, *ALF2*, *Myd88*, and *CncC*) and the reference gene (β-actin) were designed using Oligo 6.0 software and are listed in Table 1. Gene expression levels were calculated using the 2^−ΔΔCt^ method, with β-actin as the internal control. There were a total of 6 samples, and each sample was tested three times.

### 2.5. Intestinal Microflora Identification

Total genomic DNA was extracted from intestinal content samples using the TIANamp Stool DNA Kit (Tiangen Biotech, Beijing, China) according to the manufacturer’s protocols. The concentration and quality of the extracted DNA were checked using a NanoDrop 2000 spectrophotometer (Thermo Scientific, Waltham, MA, USA) and 1% agarose gel electrophoresis.

The hypervariable V3-V4 region of the bacterial 16S rRNA gene was amplified with the forward primer 338F (5′-ACTCCTACGGGAGGCAGCAG-3′) and the reverse primer 806R (5′-GGACTACHVGGGTWTCTAAT-3′). PCR reactions were performed in triplicate for each sample using TransStart FastPfu DNA Polymerase (TransGen Biotech, Beijing, China). The PCR products were purified with AMPure XP Beads (Beckman Coulter, Brea, CA, USA) and quantified using a Qubit 3.0 fluorometer (Thermo Scientific, Waltham, MA, USA).

Sequencing libraries were constructed using the Illumina DNA Prep Kit (Illumina, San Diego, CA, USA). The quality of the libraries was assessed with an ABI StepOnePlus Real-Time PCR System (Applied Biosystems, Foster City, CA, USA) and an Agilent 2100 Bioanalyzer (Agilent Technologies, Santa Clara, CA, USA).

The raw sequencing data were processed using QIIME 2 (version 2023.5). Briefly, primer sequences were trimmed, and paired-end reads were merged, quality-filtered, and denoised into amplicon sequence variants (ASVs) using the DADA2 plugin. Chimeric sequences were identified and removed. Taxonomy was assigned to the ASVs using the SILVA database (version 138). The raw sequencing data have been deposited into the NCBI Sequence Read Archive (SRA) under the accession number PRJNA1132773.

### 2.6. Non-Targeted Metabolomics Analysis

To investigate the metabolic differences between the WCM and PC systems in *E. sinensis*, non-targeted metabolomics analysis was performed on muscle tissue samples (50 mg). The analysis was conducted using a UHPLC system (1290 Infinity LC, Agilent Technologies, Santa Clara, CA, USA) interfaced with a quadrupole time-of-flight mass spectrometer (AB Sciex TripleTOF 6600, Brea, CA, USA). To achieve HILIC separation, samples were injected onto a 2.1 mm × 100 mm ACQUIY UPLC BEH Amide column with a particle size of 1.7 µm (Waters, Dublin, Ireland). The mobile phase composition, applied in both ESI positive and negative ionization modes, consisted of solvent A (25 mM ammonium acetate and 25 mM ammonium hydroxide in water) and solvent B (acetonitrile). The gradient program initiated at 95% B for 0.5 min, followed by a linear decrease to 65% B over 6.5 min, further reduction to 40% B within 1 min, and a hold at 40% B for an additional minute. This was followed by a rapid increase back to 95% B in 0.1 min, with a 3 min re-equilibration phase included to ensure column stability.

The metabolites were identified and annotated by referencing the Human Metabolome Database (HMDB) and Metlin database. Differential metabolites among groups were determined using a significance cutoff of VIP > 1 and *p* < 0.05. Subsequently, metabolic enrichment analysis and key pathway screening were carried out based on data from the Kyoto Encyclopedia of Genes and Genomes (KEGG).

### 2.7. Statistical Analyses

All data were analyzed using one-way analysis of variance (ANOVA) with SPSS software, version 20.0 (IBM Corp., Armonk, NY, USA). Unless otherwise specified, all data are expressed as mean values ± standard deviations.

## 3. Results

### 3.1. Performance Indexes

The growth performance of *E. sinensis* under the two culture systems is presented in Table 2. The final weight of the C♀ and W♀ groups was notably lower than that of the C♂ and W♂ groups (*p* < 0.05). The growth rate (GSI) of the W♀ group was significantly higher than that of the other groups (*p* < 0.05), whereas the hepatopancreas index (HIS) of the W♀ group was significantly reduced relative to the other groups (*p* < 0.05). The muscle yield (MYI) of the C♂ and W♂ groups showed a significant increase compared to the C♀ and W♀ groups (*p* < 0.05). No significant differences were observed among the groups in terms of the fullness (FNS) index, which is a comprehensive indicator for assessing the plumpness of muscle, hepatopancreas, and gonad in Chinese mitten crabs.

### 3.2. Biochemical Parameters

**Figure 2:**
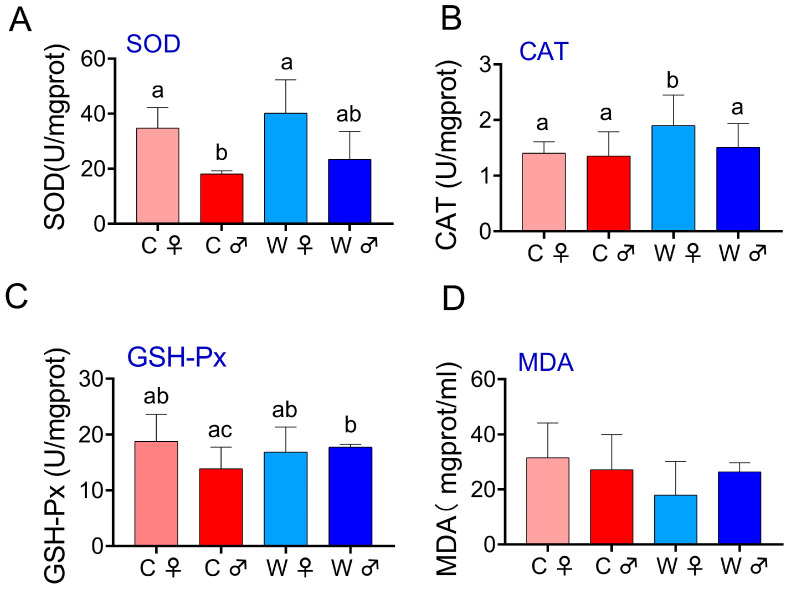
Impact of various culture methods on the hepatopancreas immune indices of *E. sinensis* (*n* = 6), SOD to total superoxide dismutase (**A**), GSH-Px to glutathione peroxidase (**B**), CAT to catalase (**C**), and MDA refers to malondialdehyde content (**D**). Note: C♀ represents control female *E. sinensis*; C♂ represents control male *E. sinensis*; W♀ denotes female *E. sinensis* in wheat-crab culture systems; W♂ denotes male *E. sinensis* in wheat-crab culture systems. Data are expressed as mean ± standard deviation. Identical letters indicate no significant difference (*p* > 0.05), while the absence of identical letters signifies a significant difference (*p* < 0.05).

In the hepatopancreas (Figure 2), SOD activity was significantly higher in the C♀ and W♀ groups compared to the C♂ group (*p* < 0.05). The activity of CAT in the W♀ group was markedly elevated compared to the other groups (*p* < 0.05). GSH-Px activity was significantly greater in the W♂ group than in the C♂ group (*p* < 0.05) but did not significantly differ between the W♀ and C♀ groups. Additionally, MDA activity levels did not significantly differ in any of the pairwise comparisons.

**Figure 3:**
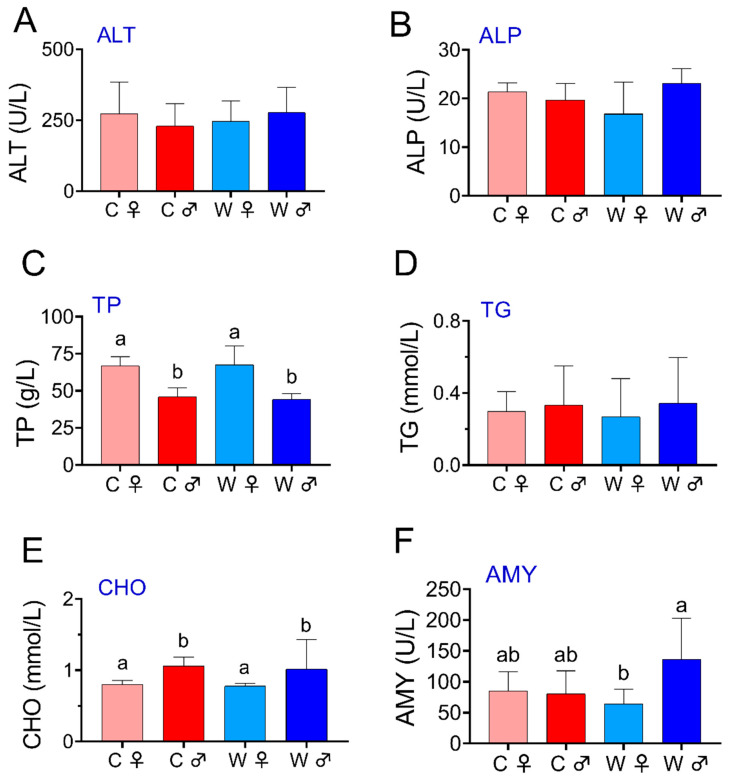
Impact of serum biochemistry on *E. sinensis* in the wheat-crab model (*n* = 6), ALT refers to alanine aminotransferase (**A**); ALP refers to alkaline phosphatase (**B**); TP refers to total protein (**C**); TG refers to triglyceride (**D**); CHO refers to total cholesterol (**E**); and AMY refers to amylase (**F**). Note: C♀ represents control female *E. sinensis*; C♂ represents control male *E. sinensis*; W♀ represents female *E. sinensis* in the wheat-crab model; W♂ represents male *E. sinensis* in the wheat-crab model. Data are expressed as mean ± standard deviation. Identical letters indicate no significant difference (*p* > 0.05), while the absence of identical letters signifies a significant difference (*p* < 0.05).

In the serum (Figure 3), the C♀ and W♀ groups exhibited higher levels of TP and CHO compared to the C♂ and W♂ groups (*p* < 0.05). However, the groups did not show significant differences in the ALT, TG, and ALP activity levels. Additionally, the W♂ group showed a significantly higher AMY content than the W♀ group (*p* < 0.05), while significant variations were not detected among the remaining groups.

### 3.3. Gene Expression Analyses

**Figure 4:**
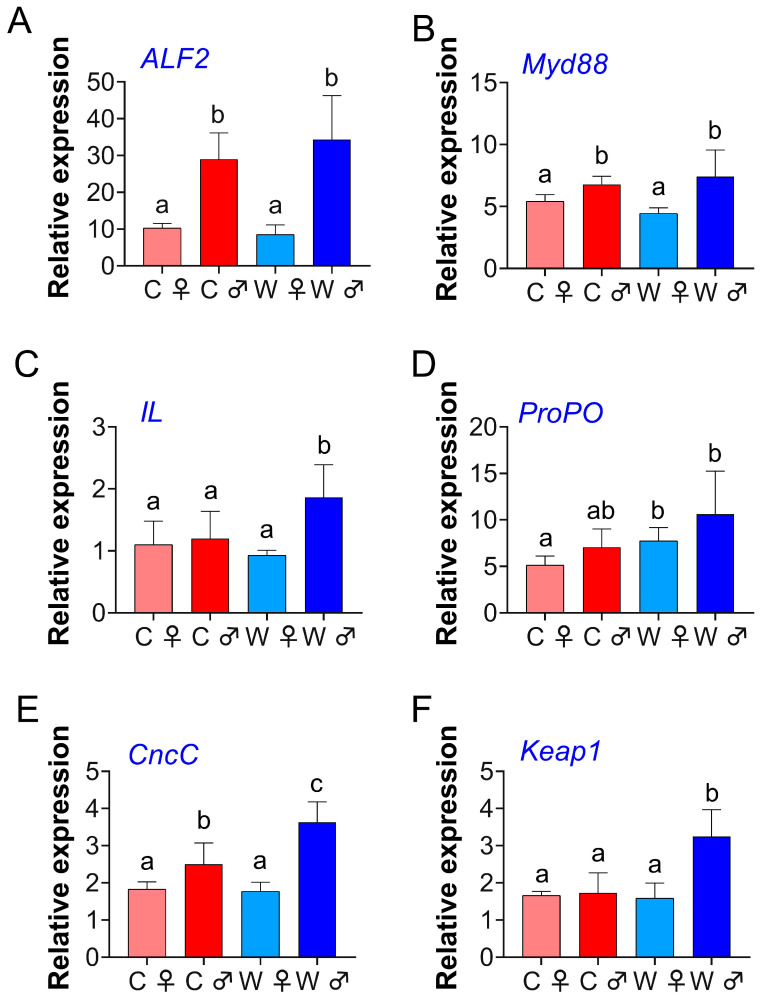
Gene expression levels in the hepatopancreas of *E. sinensis* from the wheat-crab model (*n* = 6). *ALF2* refers to anti-lipopolysaccharide factor 2 (**A**); *Myd88* refers to myeloid differentiation primary response gene 88 (**B**); *IL* refers to interleukin (**C**); *ProPO* refers to prophenoloxidase (**D**); *CncC* refers to Cap‘n’Collar Isoform C (**E**); and *Keap1* refers to kelch-like ECH-associated protein 1 (**F**). Note: C♀ denotes control female *E. sinensis*; C♂ denotes control male *E. sinensis*; W♀ denotes female *E. sinensis* in wheat-crab model; W♂ denotes male *E. sinensis* in wheat-crab model. Data are expressed as mean ± standard deviation. Identical letters indicate no significant difference (*p* > 0.05), while the absence of identical letters signifies a significant difference (*p* < 0.05).

Figure 4 shows the expression of *ALF2*, *Myd88*, and *CncC* genes in the hepatopancreas, which were markedly elevated in male crabs compared to female crabs (*p* < 0.05). The expression levels of the *IL* and *Keap1* genes were significantly higher in the W♂ group than in the other groups (*p* < 0.05). Additionally, *ProPO* gene expression was significantly upregulated under the WCM compared to the PC model (*p* < 0.05).

### 3.4. Intestinal Microbiota

β-diversity analysis indicated significant differences among the four groups (W♂, W♀, C♂, and C♀), according to an Adonis (PERMANOVA) test (genus: R^2^ = 0.2364, *p* = 0.001; OTU: R^2^ = 0.2103, *p* = 0.001; Figure S1A,B). Additionally, the alpha diversity indexes differed according to the Kruskal–Wallace test (Chao 1: *p* = 0.0016; Sobs: *p* = 0.0016; Simpson: *p* = 0.0087; Shannon: *p* = 0.0001; Figure S1C–F).

**Figure 5:**
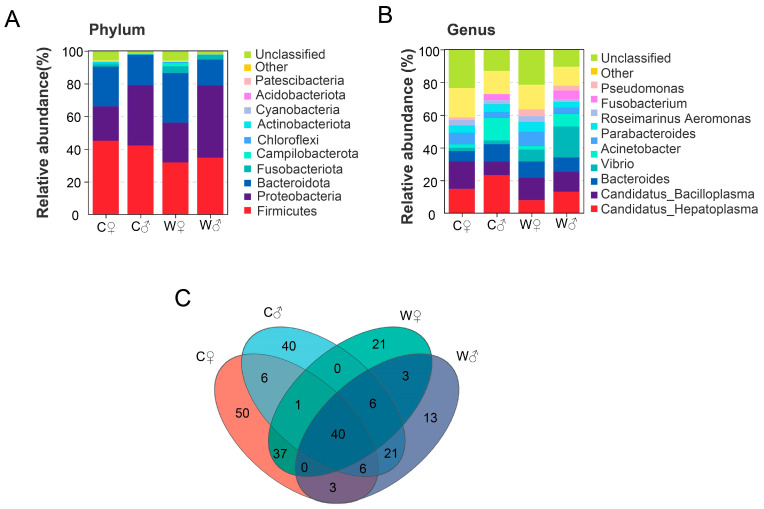
Impact of various culture patterns on the intestinal microbiota composition of *E. sinensis* (*n* = 6). Note: (**A**): Influence on microbial abundance at the phylum level; (**B**): Influence on microbial abundance at the genus level; (**C**): VENN diagram at the genus level. Note: C♀ represents control female *E. sinensis*; C♂ represents control male *E. sinensis*; W♀ represents female *E. sinensis* in the wheat-crab model; W♂ represents male *E. sinensis* in the wheat-crab model.

**Figure 6:**
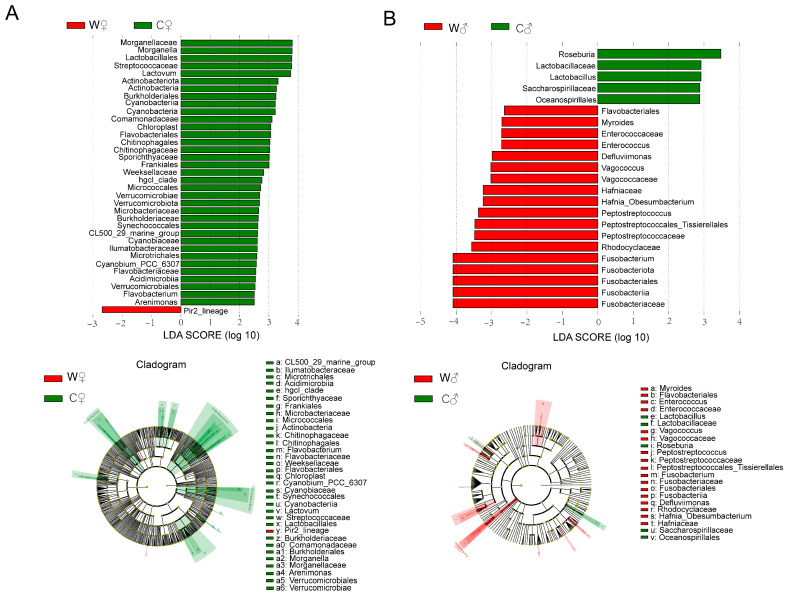
LDA scores and cladograms for the LEfSe comparison analysis between the C♀ and W♀ groups (**A**) and C♂ and W♂ groups (**B**) (*n* = 6). The bacterial taxa that showed significantly higher levels in comparisons of C♀ versus W♀ and C♂ versus W♂ are highlighted in red and green, respectively. Discriminative taxa were identified using an LDA score threshold of 2.5. Statistical significance for differences in taxonomic relative abundances was determined at a *p*-value of 0.05. Note: C♀ represents control female *E. sinensis*; C♂ represents control male *E. sinensis*; W♀ represents female *E. sinensis* in the wheat-crab model; W♂ represents male *E. sinensis* in the wheat-crab model.

Venn diagrams were constructed based on the counts of genera present in all groups. In terms of abundance, the top four phyla were Firmicutes, Proteobacteria, Bacteroidetes, and Fusobacteriota (Figure 5A); and the top nine genera were *Candidatus_Hepatoplasma*, *Candidatus_Bacilloplasma*, *Bacteroides*, *Vibrio*, *Acinetobacter*, *Paracoccus*, *Aeromonas*, *Roseimarinus*, and *Fusobacterium* (Figure 5B). The intestinal flora included 115 (C♂), 92 (W♂), 143 (C♀), and 118 (C♂) genera. Among them, 40 genera occurred in all groups, accounting for 34.19% (Figure 5C). The linear discriminant analysis (LDA) effect size (LEfSe; LDA score > 2.5, *p* < 0.05) showed that 36 microbial clades displayed statistically significant differences between the C♀ and W♀ groups (Figure 6), including *Streptococcaceae* (LDA score = 3.78), *Cyanobiaceae* (LDA score = 2.61), *Cyanobacteria* (LDA score = 3.22), *Arenimonas* (LDA score = 2.50), *Verrucomicrobiae* (LDA score = 3.69), and *Pir2_lineage* (LDA score = 2.69) (Figure 6A). Furthermore, 23 microbial clades displayed statistically significant differences between the C♂ and W♂ groups, including *Fusobacteriia* (LDA score = 4.09), *Peptostreptococcales_Tissierellales* (LDA score = 3.47), *Rhodocyclaceae* (LDA score = 3.56), *Lactobacillus* (LDA score = 3.13), and *Myroides* (LDA score = 2.70) (Figure 6B).

### 3.5. Non-Targeted Metabolomics

The impact the different culture models on the muscle metabolism of Chinese mitten crabs was analyzed using orthogonal partial least squares discriminant analysis (OPLS-DA). As shown in Figure S1, the OPLS-DA score chart of electrospray ionization (ESI) (−) (Figure S2A,B, A: C♀ vs. W♀, B: C♂ vs. W♂) showed significant separation between different groups, indicating significant differences in metabolites related to different rolling times. The arrangement test showed that the R^2^ and Q^2^ intercepts of ESI(−) were R^2^ = (0.0, 0.9), Q^2^ = (0.0, −0.17) between the C♀ and W♀ groups and R^2^ = (0.0, 0.99), Q^2^ = (0.0, 0.1) between the C♂ and W♂ groups, respectively, indicating that the OPLS-DA model is.

**Figure 7:**
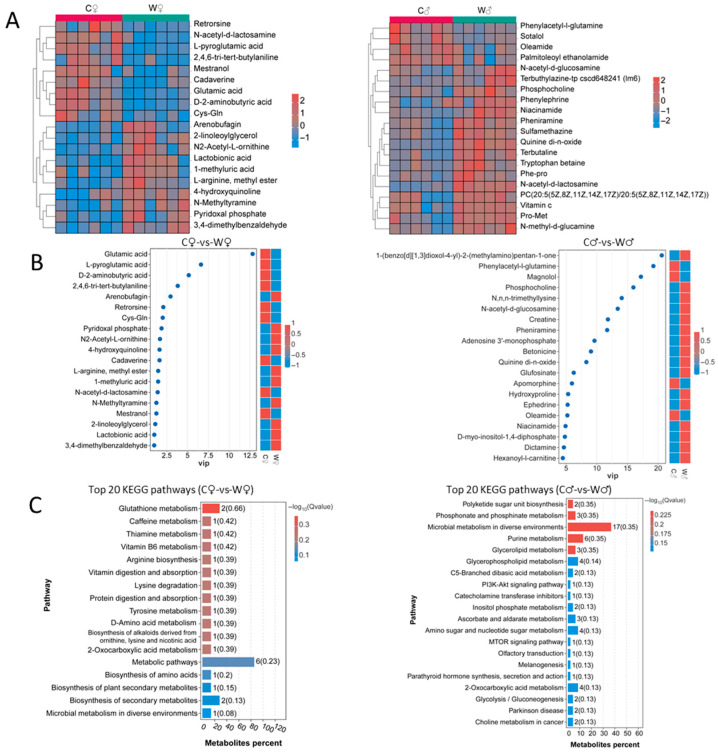
Identification and classification of differential metabolites and results of the KEGG analysis (*n* = 6). (**A**) Clustering heat map of differential metabolites. The color change from red to blue in the heat map indicates that the abundance of metabolites in each group of samples decreases from high to low. (**B**) VIP map of differential metabolites. The red rectangle on the right represents an increase, and the blue rectangle represents a decrease. (**C**) Top 20 KEGG pathways of differential metabolites. Note: The size of the squares indicates the magnitude of the impact factor, while the darker color represents a higher degree of enrichment significance. KEGG: Kyoto Encyclopedia of Genes and Genomes; C♀ represents control female *E. sinensis*; C♂ represents control male *E. sinensis*; W♀ represents female *E. sinensis* in the wheat-crab model; W♂ represents male *E. sinensis* in the wheat-crab model.

As depicted in Figure 7A,B, the heat map and VIP map clearly illustrate that the wheat-crab pattern can modify both the types and amounts of metabolites present in crab meat (VIP > 1 and *p* < 0.05). These metabolites are categorized and identified based on their chemical classifications, such as fatty acyls, triazines, carboxylic acids and derivatives, glycerophospholipids, steroids, and steroid derivatives. Furthermore, the findings indicate that N-acetyl-d-lactosamine (*p* = 0.004221), arenobufagin (*p* = 0.045766), and Cys-Gln (*p* = 0.025446) are the primary metabolites influenced by the WCM.

To gain deeper insights into the biological roles of differentially abundant metabolites, we identified signaling pathways that were significantly enriched with differential metabolites between the control group and WCM group using the KEGG database (*p* < 0.05). In the analysis between the W♀ and C♀ groups, the top 20 enriched metabolic pathways included glutathione metabolism, caffeine metabolism, thiamine metabolism, vitamin B6 metabolism, arginine biosynthesis, vitamin digestion and absorption, lysine degradation, as well as other important metabolic pathways (Figure 7C). For the comparison between the W♂ and C♂ groups, the top 20 enriched metabolic pathways included polyketide sugar unit biosynthesis, phosphonate and phosphinate metabolism, microbial metabolism in diverse environments, purine metabolism, glycerolipid metabolism, glycerophospholipid metabolism, and additional critical metabolic pathways (Figure 7C). These disrupted metabolic pathways are also strongly associated with the substantial changes observed in the aforementioned differentially abundant metabolites.

### 3.6. Correlation Analysis

**Figure 8:**
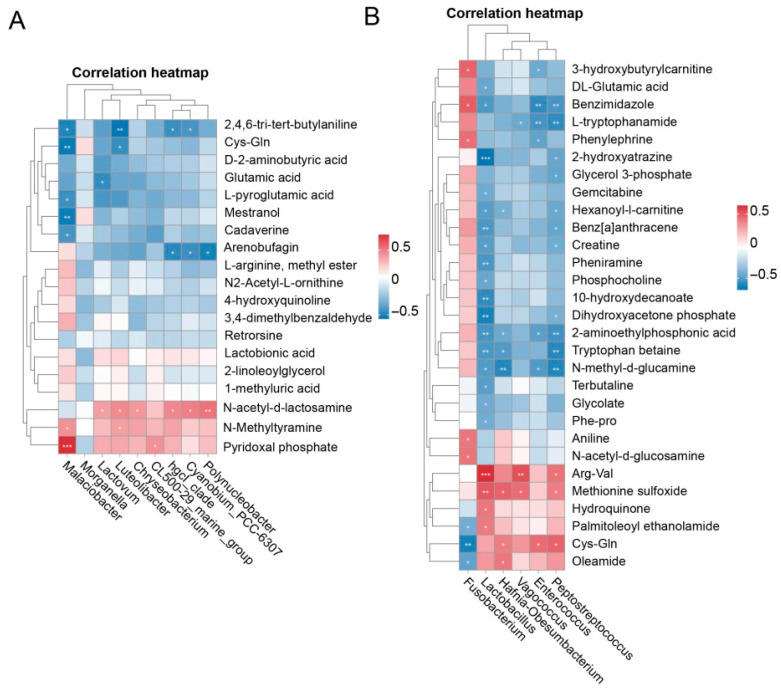
Analysis of the correlation model between microorganisms (genera) and metabolites. (**A**) W♀ vs. C♀ group, (**B**) W♂ vs. C♂ group. The colors of the squares in the figure represent the strength of the correlation. Red represents a positive correlation, and blue represents a negative correlation. “*” indicates *p* < 0.05, “**” indicates *p* < 0.01 and “***” indicates *p* < 0.001.

To explore whether the wheat crab model’s effect on muscle metabolism is linked to changes in gut microbiota, we performed a Spearman correlation analysis between differential microbial genera and metabolites (Figure 8). In the W♀ vs. C♀ group, intestinal microbiota genera were significantly correlated with N-acetyl-d-lactosamine and 2, 4, 6-tri-tert-butylaniline (Figure 8A). In the W♂ vs. C♂ group, correlations were observed with 2-aminoethylphosphonic acid and Cys-Gln (Figure 8B). Additionally, Cys-Gln was negatively correlated with Malaciobacter and Luteolibacter in the W♀ vs. C♀ group, while it was negatively correlated with Fusobacterium and positively correlated with Hafnia-Obesumbacterium, Peptostreptococcus, and Enterococcus in the W♂ vs. C♂ group. These related patterns suggest that the changes in the gut microbiota of the WCM may be associated with the alterations in the muscle metabolite profile of *E. sinensis*.

## 4. Discussion

Our study is the first to elucidate the impact of wheat-crab culture and pond monoculture on the growth performance, immunity, and gut microbiome of *E. sinensis*. Significant differences were not observed in the weight gain of *E. sinensis* between the two cultivation models, although the maturity level of *E. sinensis* under the WCM was significantly greater compared to that of the PC model. These findings suggest that the wheat-crab co-culture approach holds promise as a viable method for commercial-scale aquaculture.

### 4.1. Performance Indexes

The aquaculture environment has a significant impact on the growth performance of *E. sinensis* [22]. Environmental factors (e.g., water quality, substrate, and feed) and farming models (e.g., rice-crab, recirculating systems) directly influence crab physiology and energy allocation, ultimately affecting growth performance [23,24]. Our results indicated that the two culture models did not significantly affect the overall weight gain of *E. sinensis*, but influenced their energy allocation strategy, particularly in female crabs. A key finding was that female crabs in the WCM exhibited a significantly lower HIS alongside a significantly higher GSI compared to those in the PC model. This aligns with the typical physiological pattern during ovarian development in female crabs: nutrients stored in the hepatopancreas are mobilized and transferred to the developing ovaries, resulting in a negative correlation between GSI and HIS [25,26]. The more natural environment provided by the WCM may have promoted this physiological process, leading to earlier and more advanced gonadal development in female crabs.

More importantly, this study revealed that for many growth indices, the differences between sexes were far greater than those between the culture models. For instance, the final body weight and muscle yield of male crabs were significantly higher than those of females, a trend consistent under both culture models. This pattern clearly suggests that, for *E. sinensis*, sex is a stronger determinant of growth performance than the culture models investigated in this study. It is essential to consider sex as a critical biological variable in future aquaculture practices and related research.

### 4.2. Biochemical Parameters

In the present study, the female crab groups exhibited higher activities of CAT, SOD, and GSH-Px compared to the male crab groups; however, no significant difference was observed between the two cultivation modes. Additionally, MDA activity did not differ significantly among any of the groups. Taken together, these findings suggest that the antioxidant capacity of Chinese mitten crabs was not significantly affected by the two culture modes. Our study investigated the impact of two breeding models on the nutritional and health status of aquatic organisms, with a specific focus on TG and CHO levels. CHO plays a critical role in the synthesis of cell membranes and production of steroid hormones, with its blood concentration being affected by factors such as liver function, nutritional status, gender, and sexual development [27]. TGs serve as the primary fat storage molecules in the body and represent an important energy source for cells [28]. Our results did not show significant differences in TG and CHO levels between the two breeding models, indicating that the models did not significantly affect lipid metabolism. However, a notable gender-specific difference was observed, with male mitten crabs exhibiting significantly higher CHO levels compared to females. This finding highlights the need for further research to explore gender-specific responses and environmental responses on the nutritional and health status of aquatic organisms.

In crustaceans, hemolymph serves as an open circulatory system and plays a key role in transporting hormones, oxygen, nutrients, and other substances throughout the organism [29,30]. ALP, a glycoprotein enzyme, is recognized as a critical biomarker for assessing the condition of the plasma membrane and endoplasmic reticulum [31,32]. In this study, no significant differences in ALP activity levels were observed among the experimental groups, suggesting that the serum cells of *E. sinensis* were not affected or damaged by the ecological wheat-crab farming model. The level of serum ALT activity can serve as a tool for monitoring health status [33,34]. If liver cells are injured or their permeability increases, ALT may be released into the bloodstream, leading to elevated blood transaminase activity [35]. In our research, no significant increase in serum ALT activity was observed, suggesting that the liver cells of Chinese mitten crabs were not adversely affected by the WCM treatment.

### 4.3. Gene Expression Analyses

Innate immunity primarily depends on the recognition of pathogens by innate receptors, which modulate the expression of antimicrobial peptides by activating nuclear factor-kappa B (NF-κB) via *MyD88* [36]. Among these peptides, *ALF* is considered one of the most crucial antimicrobial compounds [37]. In this research, the expression levels of *ALF2*, *Myd88*, and *CncC* genes in the hepatopancreas did not significantly differ between the two culture models. However, male crabs showed significantly higher expression levels than female crabs, indicating that male crabs may have stronger immune defense functions. Previous studies have found that the xenobiotic transcription factor *CncC* serves as the primary regulator of antioxidant and detoxification genes [38].

The significant upregulation of *IL* and *Keap1* genes in the W♂ group indicates that male crabs have model-specific immunity and antioxidant responses. *IL* is a key cytokine in the inflammatory response, and its elevated expression indicates an elevated immune activation status in male *E. sinensis* with WCM. Meanwhile, *Keap1* is a key negative regulatory factor of the Nrf2-Keap1 pathway that regulates the expression of antioxidant genes [39]. The increase in *Keap1* expression may reflect the dynamic feedback mechanism in this pathway and could be a response to elevated oxidative stress. Female crabs may allocate a greater proportion of their energy resources to gonadal development, as demonstrated by their higher GSI, which may limit the allocation of resources to sustained and high-level immune gene expression. In contrast, male crabs do not require direct energy consumption for the formation of yolk cells, and thus may have a stronger ability to build a robust immune and antioxidant defense to cope with environmental factors. Furthermore, the expression of the *ProPO* gene, a central component of the melanization cascade, was markedly elevated in the WCM compared to the PC model. This discovery further indicates that the WCM environment has the potential to enhance the immunity and antioxidant capacity of male *E. sinensis*.

### 4.4. Intestinal Microbiota

In crustaceans, the gut serves as the habitat for a diverse range of microorganisms. It functions not only as a key organ for digestion, absorption, and nutrient exchange but also plays a crucial role in preserving host health and modulating numerous important physiological processes [40,41]. The intestinal microbiota constitute a dynamic and diverse ecosystem, where environmental and nutritional factors play a significant role in shaping the composition and abundance of the microbial community [42,43,44]. Our results provide robust evidence that both the culture model and the host sex are major drivers of microbial community structure in *E. sinensis*. We found significant differences in beta diversity among the four groups (Adonis test, R^2^ = 0.2364, *p* = 0.001), indicating distinct microbial community compositions. Furthermore, significant variations in alpha diversity indices (e.g., Chao1, *p* = 0.0016; Shannon, *p* = 0.0001) confirmed that the richness and evenness of the gut microbes were also markedly influenced between the WCM model and the PC model.

The most functionally significant alterations were observed in the abundance of specific bacterial taxa. At the phylum level, the predominant bacterial groups in crabs across all four groups were Firmicutes, Proteobacteria, Bacteroidetes, and Fusobacteriota [45,46]. Among them, Firmicutes are key contributors to energy harvest and short-chain fatty acid production, which enhance host nutrient absorption and fortify intestinal barrier integrity, thereby exerting anti-inflammatory effects [47]. At the genus level, the LEfSe identified specific biomarkers indicative of the culture environment. Notably, the class *Fusobacteriia*—a taxon often associated with inflammatory conditions in aquatic animals—was identified as a significant biomarker in the control male group (C♂). In stark contrast, the WCM male group (W♂) was prominently enriched with *Lactobacillus*, a genus widely recognized for its probiotic properties, including pathogen inhibition and immunomodulation [48]. Furthermore, both *Candidatus_Hepatoplasma* and *Candidatus_Bacilloplasma* belong to the phylum Tenericutes. Several studies have indicated that *Candidatus_Bacilloplasma* is commonly found in healthy crustaceans [49]. These findings indicate that the WCM leads to alterations in the composition of gut microbiota, characterized by an increase in beneficial microorganisms and a reduction in harmful ones. These findings align with previous studies on integrated culture systems for *E. sinensis*, such as the rice-crab model, where similar enrichments of beneficial bacterial consortia have been reported [25]. We propose that the WCM environment, through the provision of complex organic matter from wheat residues and a diverse environmental microbiome, acts as a continuous source of prebiotic substrates and beneficial microbial inocula. This ecological feature selectively enriches a gut microbiota that is more metabolically efficient and resilient against pathogen colonization, thereby contributing to the enhanced immune competence and overall physiological well-being observed in crabs reared under the WCM model.

### 4.5. Non-Targeted Metabolomics

In order to examine the effects of the two culture models on the muscle metabolism of *E. sinensis*, we employed OPLS-DA for non-targeted metabolomics analysis. The results indicated significant differences in metabolic pathways between female crabs (C♀ and W♀) and male crabs (C♂ and W♂), which may have important implications for their immune and metabolic functions.

In our study, the significant enrichment pathways of differential metabolites included glutathione metabolism, arginine biosynthesis, vitamin digestion and absorption, thiamine metabolism, and vitamin B6 metabolism in the C♀ and W♀ groups. Earlier research has demonstrated that glutathione strengthens the antioxidant defense and oxidative stress response in crabs, safeguarding them against potential harm [50]. Additionally, dietary arginine plays a role in modulating the expression of genes related to antioxidation and immunity in crabs, thereby mitigating T-2 toxin-induced damage [51]. Thiamine and vitamin B6 metabolism are the basis for maintaining normal nervous system function and energy metabolism, and the normal conduct of these processes is also important for the maintenance of immune function under the wheat-crab treatment.

In contrast, the significant enrichment pathways of differential metabolites included polyketide sugar unit biosynthesis, phosphonate and phosphinate metabolism, purine metabolism, glycerolipid metabolism, and glycerophospholipid metabolism between the C♂ and W♂ groups. For example, glycerolipid and glycerophospholipid metabolism are essential for preserving the stability and fluidity of cell membranes, which directly impact the proper functioning of immune cells and efficiency of substance transport [52]. Moreover, purine metabolism is intricately connected to energy production and signal transduction pathways, potentially influencing immune function by modulating intracellular signaling networks [53,54]. Additionally, processes such as polyketide biosynthesis may play a role in the growth and development of male crabs [55,56], specifically by affecting their physiological traits through changes in cellular structure and metabolic activity.

In addition, omics correlation analysis serves as a vital tool for elucidating the relationship between gut microbiota patterns and metabolite changes [57]. The correlation analysis between gut microbiota and metabolites provides compelling evidence for functional crosstalk between microbial communities and host metabolism. The significant associations between specific microbial genera (e.g., *Fusobacterium*, *Lactobacillus*) and muscle metabolites suggest that the WCM-induced gut microbiota restructuring serves as an upstream regulator of crabs’ metabolic phenotypes. We propose that the altered gut microbial community modulates host metabolism through several interconnected mechanisms: the production of microbial metabolites that directly influence metabolic pathways in crabs, enhanced degradation and utilization of dietary components, thereby altering nutrient availability. This microbiota–metabolite axis constitutes a pivotal mechanism by which the WCM environment translates ecological advantages into improved physiological performance, offering a comprehensive framework for understanding how integrated culture models enhance crab health through coordinated microbiome–metabolome interactions.

## 5. Conclusions

This study provides the first comprehensive evaluation of the WCM as a sustainable alternative to the PC for *E. sinensis*. Our results demonstrate that while the WCM does not enhance final body weight, it induces significant physiological improvements characterized by sexual dimorphism: females exhibit accelerated ovarian maturation, while males show enhanced immune and antioxidant capacities through upregulation of key genes (*IL*, *Keap1*, *ProPO*). The health benefits of WCM are fundamentally supported by a restructuring of the gut microbial ecosystem, marked by increased beneficial Firmicutes and *Lactobacillus* alongside reduced potential pathogens. These microbial changes are functionally linked to systemic metabolic reprogramming, as revealed by non-targeted metabolomics, indicating improved antioxidant defense in females and optimized energy metabolism in males. In conclusion, the WCM successfully translates its ecological advantages into measurable health benefits for *E. sinensis*. This integrated approach not only enhances crab quality but also aligns with national food security strategies by enabling simultaneous grain and aquaculture production on the same land. We recommend WCM as a sustainable practice that supports both ecological integrity and economic viability in Chinese mitten crab aquaculture.

## Figures and Tables

**Table 1:** Primer sequences utilized for real-time PCR analysis.

Gene	Primer Sequence Forward (5′-3′)	Primer Sequence Reverse (5′-3′)	Tm (°C)	Length (bp)
*ALF2*	TCGGCAAAGTAACTGAAACTCTG	TAATGAGGCGGGGTGACAAG	60.5	90
*Myd88*	GTGGGTCAGCTGTGGAACTAT	TCCCTGGCTTACAACCTTGTC	59.7	105
*ProPO*	GTGACCGTCCGCATCTTCAT	TGTGCCTGGCTTCAATGTGT	60.5	123
*IL*	CGATCCTACGAGTTCTTCA	GCACTTGGTGTTGTCATC	59.5	125
*Keap1*	CTTCATGTACACTGGCGAGA	GTACAGCAAGCGTCAATCACA	61.0	105
*CncC*	GCATCCTTCTGGTACCTCGTT	CACTGCTTTGGCTCATCCTTG	58.5	91
*β-actin*	TCGTGCGAGACATCAAGGAAA	AGGAAGGAAGGCTGGAAGAGTG	59.5	178

**Table 2:** Growth performance and tissue indexes of *E. sinensis* in the wheat-crab co-culture system.

Groups	Weight (g)	HIS (%)	GSI (%)	MYI (%)	FNS (%)
C♀	166.92 ± 24.11 ^a^	8.43 ± 1.02 ^a^	4.45 ± 1.09 ^b^	26.53 ± 2.31 ^a^	0.43 ± 0.01 ^a^
C♂	220.75 ± 43.97 ^b^	8.67 ± 1.35 ^a^	2.86 ± 0.41 ^a^	29.41 ± 1.68 ^b^	0.46 ± 0.04 ^a^
W♀	167.30 ± 25.06 ^a^	7.68 ± 0.81 ^b^	5.59 ± 2.07 ^b^	26.63 ± 1.45 ^a^	0.43 ± 0.02 ^a^
W♂	221.43 ± 38.49 ^b^	8.01 ± 1.07 ^a^	3.37 ± 0.53 ^a^	29.08 ± 2.04 ^b^	0.47 ± 0.03 ^a^

Note: C♀: female *E. sinensis* in the pond culture model; C♂: male *E. sinensis* in the pond culture model; W♀: female *E. sinensis* in the wheat-crab model; W♂: male *E. sinensis* in the wheat-crab model; FNS: fullness index; MYI: muscle yield index; HIS: hepatopancreas index; GSI: gonadosomatic index. Comparisons were performed between crabs of the same sex between the two model treatments, with a indicating *p* > 0.05 (non-significant) and b indicating *p* < 0.05.

## Data Availability

The raw data supporting the conclusions of this article will be made available by the authors on request.

## Supplementary Materials

The following supporting information can be downloaded at: https://www.mdpi.com/article/10.3390/microorganisms13102396/s1, Figure S1: Impact of different culture patterns on beta diversity indices (A,B) and alpha diversity indices (C–F) of intestinal microbiota (*n* = 6). Note: C♀ represents control female *E. sinensis*; C♂ represents control male *E. sinensis*; W♀ represents female *E. sinensis* in wheat-crab model; W♂ represents male *E. sinensis* in wheat-crab model; Kru-Wall: Kruskal-Wallis test. Figure S2: Quality assessment of metabolomic data for comparisons between C♀ vs W♀ and C♂ vs W♂. Score plot of orthogonal partial least squares discriminant analysis (OPLS-DA) (A: C♀ vs W♀, B: C♂ vs W♂) and permutation test results (C: C♀ vs W♀, D: C♂ vs W♂) in negative ion mode (*n* = 6). Note: C♀ represents control female *E. sinensis*; C♂ represents control male *E. sinensis*; W♀ represents female *E. sinensis* in wheat-crab co-culture systems; W♂ represents male *E. sinensis* in wheat-crab co-culture systems.
