# Supplementary material for: Effects of the Wheat Crab Model and the Pond Culture Model on the Growth, Metabolism and Intestinal Microbiota of the Chinese Mitten Crab (Eriocheir sinensis)
Source: Microorganisms. 2025 Oct 19;13(10):2396. doi: 10.3390/microorganisms13102396 (PMC12566014; doi:10.3390/microorganisms13102396)
Supplement: Supplementary file 1 [file microorganisms-13-02396-s001.zip › microorganisms-3878138-supplementary.pdf]

Wheat-crab cultivation results in earlier maturation and better health of female crabs (*Eriocheir sinensis*)

Yang Min[a]*,Ling Jun*[a]*, Li Tong*[a]*, Yu Chengchen*[a]*, Jiang He*[a]*, Pan Tingshuang*[a*]

[a]Anhui Key Laboratory of Aquaculture and Stock Enhancement & Fishery Institute of Anhui Academy of Agricultural Sciences, Hefei 230041, China

[*]These authors contributed equally to this work.

Email addresses:

Yang Min: yangmin831001@163.com

Ling Jun: fisherlling@163.com

Li Tong: little_li_tong@163.com

Yu Chengchen: ccyu549@163.com

He Jiang: hfjianghe@sina.cn

[*]**Corresponding author:**

Pan Tingshuang. Tel: 18919646705, E-mail: pantingshuang@163.com

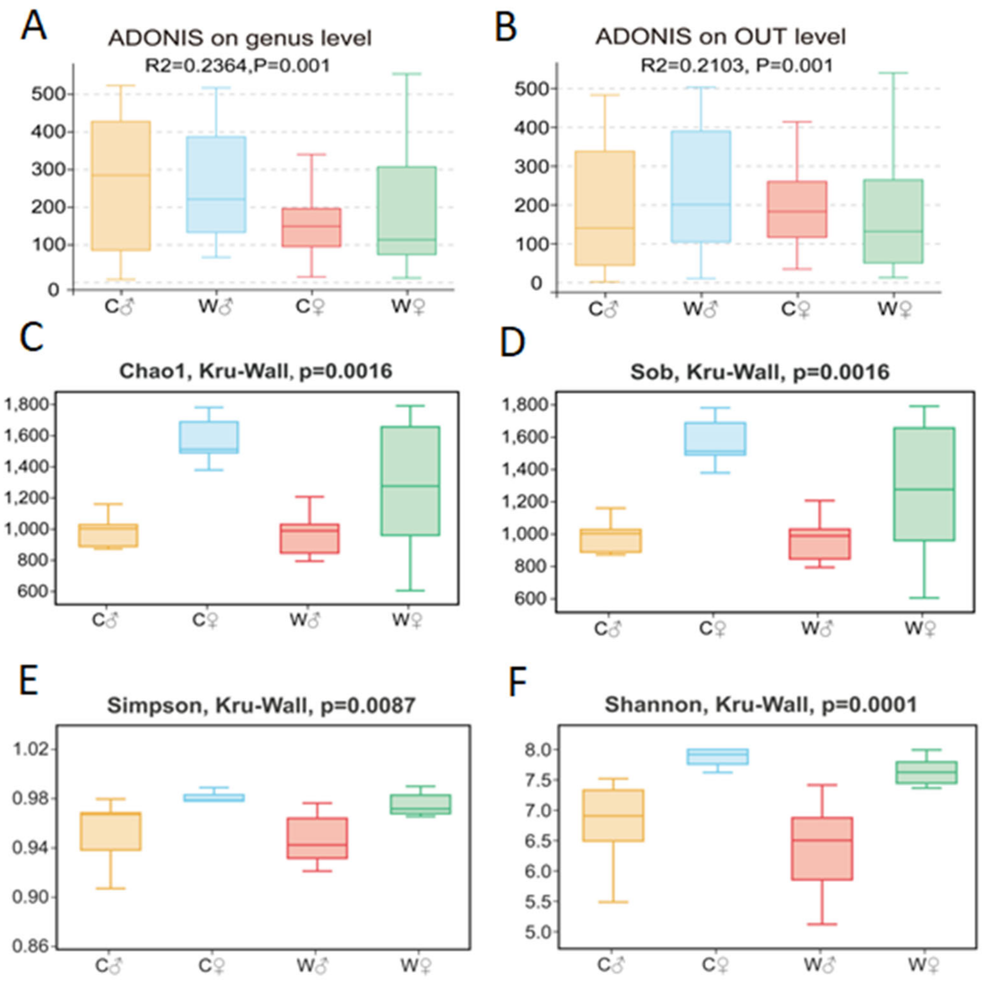

Figure S1. Impact of different culture patterns on beta diversity indices (A and B) and alpha diversity indices (C, D, E, and F) of intestinal microbiota (*n* =6). Note: C♀ represents control female *E. sinensis*; C♂ represents control male *E. sinensis*; W♀ represents female *E. sinensis* in wheat-crab model; W♂ represents male *E. sinensis* in wheat-crab model; Kru-Wall: Kruskal-Wallis test.

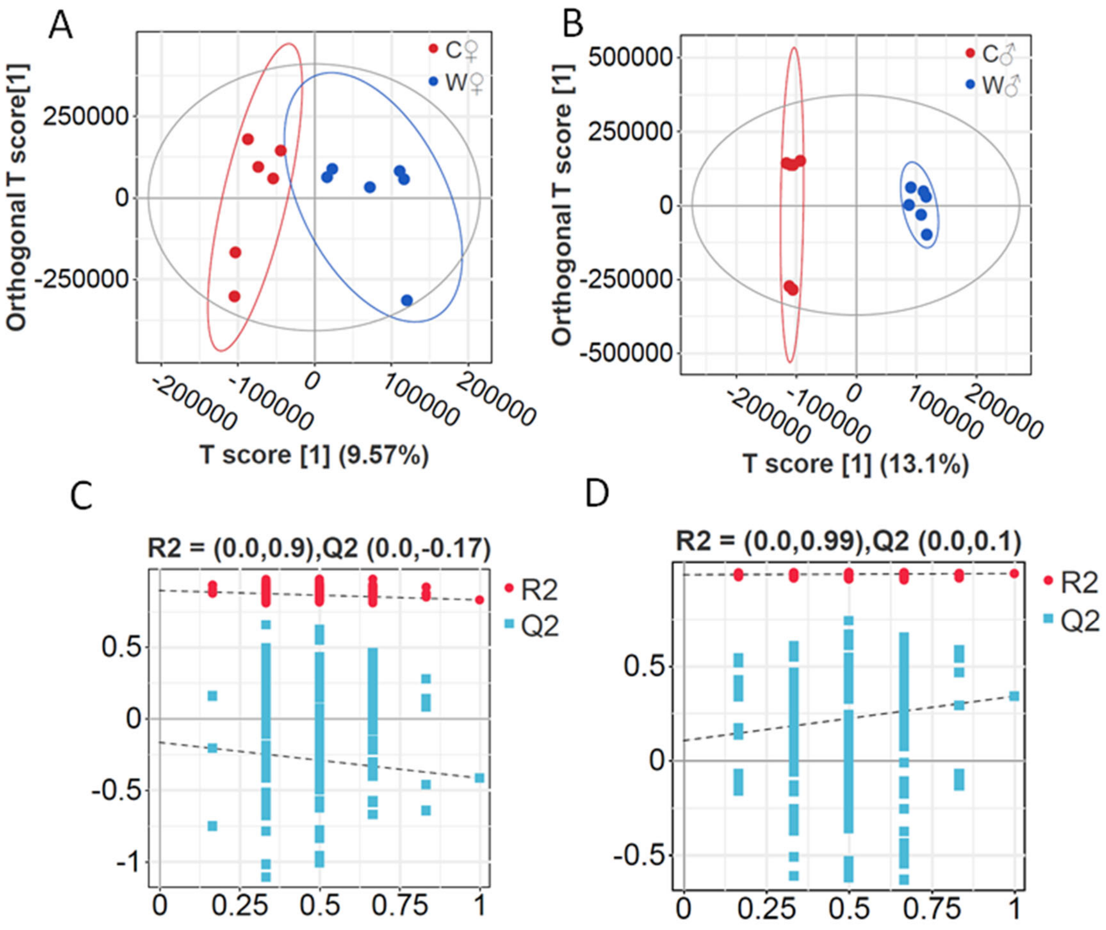

Figure. S2 Quality assessment of metabolomic data for comparisons between C♀ vs W♀ and C♂ vs W♂. Score plot of orthogonal partial least squares discriminant analysis (OPLS-DA) (A: C♀ vs W♀, B: C♂ vs W♂) and permutation test results (C: C♀ vs W♀, D: C♂ vs W♂) in negative ion mode (n =6). Note: C♀ represents control female *E. sinensis*; C♂ represents control male *E. sinensis*; W♀ represents female *E. sinensis* in wheat-crab co-culture systems; W♂ represents male *E. sinensis* in wheat-crab co-culture systems.
